# An Investigation into the Remarkable Medicinal Effects Resulting from the External Application of Veratria

**Published:** 1834-04-01

**Authors:** 


*An Investigation into the remarkable Medicinal Effects resulting from the External Application of Veratria.* By ALEXANDER TURNBULL, M.D.—London. 8vo. pp. 96.

WE are informed that the Father of Physic increased his knowledge of the art which has immortalized his name, by reading those tablets suspended in the temples of the gods, on which the grateful sick had recorded the nature of their disease, and the medicine by which it was cured. So useful is the honest history of a case, even when fidelity lacks the perfection which knowledge alone can impart! But far more useful does the register of disease become when it is drawn up by one who is qualified for his task, not only by honesty, but by science; who can select the most instructive from a crowd of cases; and who, like an old general, engages our sympathies in his successes, by the candour with which he narrates his defeats. There is a short passage in Celsus relating to this last point, which is at once so just and so elegant, that we cannot refrain from quoting it. "A suturis se deceptum esse, Hippocrates memoriæ prodidit; more scilicet magnorum virorum, et fiduciam magnarum rerum habentium. Nam levia ingenia, quia nihil habent, nihil sibi detrahunt: magno ingenio, multaque nihilominus habituro, convenit etiam simplex erroris confessio; præcipueque in eo ministerio, quod utilitatis causâ posteris traditur; ne qui decipiantur eâdem ratione, quâ quis ante deceptus est." (*De Medicinâ,* lib. viii. cap. 4.) Great men, says the classic physician, who are conscious that they have done great things, can afford to confess that they have made a mistake; but your little people, having nothing, will give up nothing, and are unceasingly in the right. Alas! at present we have to do with one who will give up nothing—nihil sibi detrahit.

Every one knows that, in its native or natural combinations, as in colchicum and veratrum, veratria has been used, and to some extent, in this country, but always cautiously, from its known power of irritating the mucous membrane; and the latter plant is very seldom given in the present day, from its pre-eminent tendency to stimulate such surfaces. The French physicians were the first to use veratria, as well as most of the other active principles of plants, for the knowledge of which we are indebted to the researches of modern chemistry. Magendie, in his "Formulaire," mentions several modes of employing it, and states the required strength for the ointment to be four grains to the ounce of axunge. Our author, we suppose, looks upon this as rather milksop practice; for he recommends an ointment of from fifteen to twenty grains to the ounce; and of this about one tenth is to be applied by friction over the part affected, once, twice, or more times in the day. Instead of causing any bad consequences, he tells us that its action is quite salutary.

"The first circumstance which must strike every person who prescribes the external uses of this medicine, is the very remarkable difference which is found to exist betwixt its effects upon the body when so applied, and those which result from its external exhibition. We have seen that, when applied to any of the mucous membranes, even in the smallest quantity, it produces the most violent irritation, and that when rubbed upon the surface of the body to the extent of six or eight grains a day, for several weeks, or even months together, no such consequences follow; for although the constitution has evidently, during the greater part of the time, been under the influence of the veratria, so far from acting in that manner, it has been observed to calm irritation, remove pain, and produce considerable elevation of spirits. The general health and appearance begin to improve, the appetite remains unimpaired, or even becomes better, the patient experiences not the slightest degree of nausea; and the bowels, instead of being acted upon in the manner in which the internal exhibition of the medicine would lead us to anticipate, are either altogether unaffected, or such a degree of constipation is induced as to render the use of purgatives necessary to keep them in their usual state." (P. 5.)

After giving us a few more observations upon the very different effects of veratria, when rubbed upon the skin, to those which are commonly observed when it is given as an internal remedy, Dr. Turnbull warns us to be careful in obtaining the alkaloid quite pure; for we may be sure that it is adulterated if it should fail in affording the relief which we had anticipated.

The remarks which follow, we suppose are intended to deprecate criticism, and to prepare us for believing all that our author chooses to relate of the wonderful powers of his favourite medicine.

"In an inquiring age like the present, it behoves an individual, in laying before the profession any new plan of treatment, especially if that be applicable to diseases which have heretofore been considered either very obstinate or incurable in their nature, not to say more in its favour than the facts brought to light during its investigation warrant; for experience teaches us that many remedies, the prudent use of which might have rendered the most essential service to medical science, have suffered, often irremediably, in consequence of the rash and inconsiderate praises heaped upon them by their discoverers." (P. 9.)

This is very true; but we would ask our author whether he does not conceive that he is falling into the very error he decries, by giving us whole hosts of cases, which he himself allows may be considered "as savouring too much of the marvellous." The plain truth we believe to be, that this book was never intended for the use of the professional reader, but for the profane laity; for the details of the cases are so loose, and so vague, as to be of not the slightest service to the practitioner. There is a very large class of persons in this kingdom who are totally unconnected with medicine, either theoretically or practically, but who make it a point to peruse every work upon medicine which falls in their way, more especially when enlivened by the relation of cases: these idlers are a species of perambulating advertisements, and it is for this class that we infer this "Investigation" was penned; for its whole tenor is of the kind with which these small wits delight to regale themselves: they will relate the prodigies performed by the new medicine to all their valetudinary acquaintances, and will doubtless add a few piquant relishes from their own fancy, exemplifying the old adage, that a story does not lose by the telling.

It is almost cruel, perhaps, to subject a work intended for the loungers of Bath and Cheltenham to medical criticism; and a grave examination of a pump-room book may be considered quite as supererogatory as if a chemist were to shew, without sparing us a single test, that tinsel is not gold. Yet we will venture to trespass a little longer on the patience of our readers, and unfold some of the properties of the Panacea Turnbulliana.

The principal diseases in which Dr. Turnbull has used the veratria ointment are rheumatism, gout, ascites, tic, or neuralgia, dysmenorrhœa, ovarian dropsy, paralysis, and diseases of the heart. This is a very tolerable number of maladies for one remedy to cure radically, and we congratulate both our profession and the world generally upon the discovery. How grateful ought the poor patient to be, who is suffering from diseased heart, to the man who has found out an ointment which, by only being rubbed twice or thrice upon the part, removes the complaint, and compels the impaired organ to reassume its healthy action. (See Cases 1, 3, and 4.) In Case 7, the patient is described as being sixty years of age, and suffering from violent beating of the heart, strong pulsation in the neck, throbbing and giddiness in the head, and a continual whizzing noise in the left ear; to these were added considerable anxiety, sleep interrupted by palpitation, and pain in the region of the heart. Case 9 is nearly similar in its details, but the symptoms were more aggravated. Your ordinary doctor, now, would have given up these patients; but then your ordinary doctor is not a Turnbull. In both instances the whole of the bad symptoms were instantly relieved by the first application of the ointment, and entirely subdued after it had been used only three times.

Neuralgia, or tic, would seem to be a more sturdy opponent to the powers of the alkaloid than most other complaints; for it required much larger doses to be used for its dislodgment than had been called for in any of the previously mentioned diseases. The author is accustomed, in cases of long duration, to apply an ointment over the pained part twice as strong as that in common use, with the express purpose of getting the system as rapidly as possible under the influence of the remedy. The Ung. Turnbull. fort. contains forty grains of veratria to the ounce of axunge. The directions to be observed are, that the part suffering is to be rubbed for fifteen or twenty minutes, "until the heat and tingling caused by the friction have been so great as to produce an impression on the feelings of the patient equal to that arising from the disease itself." We are led to infer from this that the heat and tingling are to be looked upon merely as signs of the remedy having began to take effect, being otherwise too trivial to excite a moment's consideration. We, however, happen to know a medical gentleman, of considerable eminence in his profession, who, having heard of the utility of the veratria in rheumatism, and being at the time afflicted with it in one of his arms, was induced to rub some of the weaker ointment (twenty grains to the ounce) over the part: soon after its application, the heat and tingling were not only excessive, but intolerable, until at length he was obliged to take opium to produce even anything approaching to a state of quietude.

The next morning, to his great annoyance and astonishment, he found his arm covered by an eczematous eruption, and, what was worse than all, the rheumatism remained unabated. According to Dr. Turnbull, however, the same magical consequences are produced by this remedy in this as in other painful diseases; one, or at most two, rubbings having proved sufficient to banish the pain, and cure the patient: and this is the more surprising, as, in some of the cases, the malady had been existing for a lengthened period of time,—in one, for instance, for thirty-six years, in a second for twenty-two, in a third for sixteen, and in several others for eleven, nine, eight, seven, five, and four years; and yet all were cured by once or twice rubbing.

If it be productive of the happiest effects in acute rheumatism, our ointment outdoes itself in chronic attacks, and restores stiffened joints to their natural and legitimate uses.

We really must hasten to terminate our remarks, for we are perfectly sated with these therapeutical wonders,—these tales in which the fairy Veratria puts to flight a hundred gigantic diseases.

In concluding, we shall only remark, that veratria seems to be endowed with the faculty of instinct, and that it selects the proper cases in which it shall produce its peculiar action very rapidly; while, again, in others, it allows a continuance of its application without causing any other sensations than those of gratification and pleasure. In fact, in some parts of this book we are told that twice, and at most thrice rubbing, will cure the most frightful complaints; whilst in others, the author says, he has ordered patients to continue its use for four months together, without any intermission; in others, he has given them a "carte blanche" to employ it how, when, and where they please.

We should suggest to Dr. Turnbull,—we presume not to advise,—that, when he next writes upon any subject, either a new remedy or a new disease, that he should give to the world not only an account of his success, but also of his failures; for he may rest assured that not only physicians, but the thinking portion of the unprofessional world, will look with great suspicion upon any drug, however powerful, or mode of treatment, however serviceable, which comes before them in such a questionable shape as he has presented his veratria; and, as he himself says, in a paragraph we have quoted at the commencement of this article, "it behoves an individual, in laying before the profession any new plan of treatment, not to say more in its favour than the facts brought to light during its investigation warrant:" it is a pity he has not taken his own advice; for we more than suspect that the remedy has not yet been discovered which enjoys such miraculous powers as are said to be possessed by the veratria in the hands of Dr. Turnbull.

